# Association of insomnia with incident dementia – considering the competing risk of death

**DOI:** 10.1002/alz.095212

**Published:** 2025-01-09

**Authors:** Sonja Sulkava, Jari Haukka, Tiina Paunio

**Affiliations:** ^1^ Finnish Institute for Health and Welfare, Helsinki, Uusimaa Finland; ^2^ University of Helsinki, Helsinki, Uusimaa Finland

## Abstract

**Background:**

Sleep disturbances have been suggested as potential risk factors for neurodegenerative diseases. However, longitudinal studies on insomnia and dementia have mostly had too short follow‐up time to separate etiological risk factors from prodromal symptoms. Moreover, the impact of mortality as a competing risk has been overlooked. This study aims to clarify the relationship between insomnia symptoms and dementia incidence, considering the competing risk of death, within a large cohort from the Finnish population.

**Method:**

The National FINRISK study, collected every five years from 1972 to 2012, included 63 013 Finns with baseline data. Approximately 12% (7638) developed dementia according to the health care registers. The follow‐up time varied from 7 to 47 years (mean = 25 years). We studied association of self‐reported insomnia (never, sometimes, often) at baseline with dementia incidence using in parallel Poisson model and Fine‐Gray model accounting for the competing risk of death. Additional analysis directly examined this competing risk. Study year, follow‐up time, sex, education, and cardiovascular risk factors were adjusted for. Sensitivity analyses included individuals with over 10 years of follow‐up.

**Result:**

In the Poisson model, insomnia symptoms “sometimes” (IRR = 1.10[1.05‐1.16]) and “often” (IRR = 1.18[1.09‐1.28]) showed significant association with incident dementia and a dose‐response relationship. The results survived the sensitivity analyses. In the Fine‐Gray model, insomnia “sometimes” (HR = 1.07[1.02‐1.12) but not “often” (1.07[0.99‐1.16]) showed significant associations. Significant associations with the competing risk of death surpassing that of dementia were detected for both categories with IRRs of 1.12[1.09‐1.15]) and 1.32[1.27‐1.37]), respectively.

**Conclusion:**

The findings from the Poisson model suggest that insomnia may serve as an etiological risk factor for dementia. However, the Fine‐Gray model, which focuses on the incidence of dementia in the presence of competing risk, did not show significant association with insomnia “often”. This suggests that individuals with insomnia face a higher risk of death before potentially developing dementia, implying that insomnia might not increase the true dementia incidence, despite being a risk factor. These results highlight the significance of including the competing risk of death in dementia epidemiological studies to fully understand the associations with risk factors.

